# E7449: A dual inhibitor of PARP1/2 and tankyrase1/2 inhibits growth of DNA repair deficient tumors and antagonizes Wnt signaling

**DOI:** 10.18632/oncotarget.5846

**Published:** 2015-10-20

**Authors:** Sharon McGonigle, Zhihong Chen, Jiayi Wu, Paul Chang, Donna Kolber-Simonds, Karen Ackermann, Natalie C. Twine, Jue-Lon Shie, Jingzang Tao Miu, Kuan-Chun Huang, George A. Moniz, Kenichi Nomoto

**Affiliations:** ^1^ Discovery Biology, Oncology PCU, Eisai Inc., Andover, MA 01810, USA; ^2^ Integrated Chemistry, Eisai Inc., Andover, MA 01810, USA; ^3^ Department of Biology, Massachusetts Institute of Technology, Cambridge, MA 02139, USA; ^4^ Koch Institute for Integrative Cancer Research, Massachusetts Institute of Technology, Cambridge, MA 02139, USA; ^5^ Current address: Moderna Therapeutics, Cambridge, MA 02139, USA; ^6^ Current address: Biogen, Cambridge, MA 02142, USA

**Keywords:** E7449, PARP, tankyrase, inhibitor, Wnt

## Abstract

Inhibition of Poly(ADP-ribose) Polymerase1 (PARP1) impairs DNA damage repair, and early generation PARP1/2 inhibitors (olaparib, niraparib, etc.) have demonstrated clinical proof of concept for cancer treatment. Here, we describe the development of the novel PARP inhibitor E7449, a potent PARP1/2 inhibitor that also inhibits PARP5a/5b, otherwise known as tankyrase1 and 2 (TNKS1 and 2), important regulators of canonical Wnt/β-catenin signaling. E7449 inhibits PARP enzymatic activity and additionally traps PARP1 onto damaged DNA; a mechanism previously shown to augment cytotoxicity. Cells deficient in DNA repair pathways beyond homologous recombination were sensitive to E7449 treatment. Chemotherapy was potentiated by E7449 and single agent had significant antitumor activity in BRCA-deficient xenografts. Additionally, E7449 inhibited Wnt/β-catenin signaling in colon cancer cell lines, likely through TNKS inhibition. Consistent with this possibility, E7449 stabilized axin and TNKS proteins resulting in β-catenin de-stabilization and significantly altered expression of Wnt target genes. Notably, hair growth mediated by Wnt signaling was inhibited by E7449. A pharmacodynamic effect of E7449 on Wnt target genes was observed in tumors, although E7449 lacked single agent antitumor activity *in vivo*, a finding typical for selective TNKS inhibitors. E7449 antitumor activity was increased through combination with MEK inhibition. Particularly noteworthy was the lack of toxicity, most significantly the lack of intestinal toxicity reported for other TNKS inhibitors. E7449 represents a novel dual PARP1/2 and TNKS1/2 inhibitor which has the advantage of targeting Wnt/β-catenin signaling addicted tumors. E7449 is currently in early clinical development.

## INTRODUCTION

Poly(ADP-ribose) Polymerases (PARPs) catalyze the post-translational modification of proteins through addition of ADP-ribose, using nicotinamide adenine dinucleotide as substrate [1]. The PARP family comprises 17 members, identified through sequence homology to the PARP1 catalytic domain. PARP1 and 2 and PARP5a and 5b (also known as tankyrase1 and 2; TNKS1 and 2) catalyze the addition of poly(ADP-ribose) (PAR), whereas the majority of family members incorporate single ADP-ribose units to substrate proteins [2, 3]. Covalent modification by the addition of PAR serves to regulate the function of target proteins, which often include the PARP enzymes themselves [4]. Large, linear and/or branched chains of PAR recruit binding proteins and serve as a scaffold for generation of large protein complexes [5, 6, 7].

PARP family enzymes are involved in many physiological processes, including cell division, regulation of transcription, maintenance of telomere integrity, control of protein degradation, and cell survival and death [8, 9]. Additional important functions in cellular stress responses include detection of DNA damage, DNA repair, response to heat shock, response to unfolded protein in the endoplasmic reticulum, and the cytoplasmic stress response [6, 8, 10, 11]. Discovered more than 40 years ago, PARP1 is the founding member and the best characterized PARP [12, 13]. It is considered a significant anticancer target due to its important function in DNA damage repair and the maintenance of genomic stability as well as additional functions in transcriptional regulation and epigenetics [9, 14]. PARP1 is a highly abundant nuclear enzyme that is recruited to and activated by sites of DNA damage. PARP2 is similarly mobilized; however PARP1 activity is responsible for the majority (90–95%) of PAR generated by genotoxic stress [15, 16]. PARP1 is involved in repair of single strand DNA breaks via the base excision repair (BER) pathway and non-homologous end joining (NHEJ) pathways [15, 17]. PARP1 inhibition in cancers defective in homologous recombination (HR) repair such as those containing mutations in *BRCA1* or *BRCA2*, leads to effective killing through synthetic lethality [18, 19]. In addition, preventing DNA repair through inhibition of PARP1/2 sensitizes tumor cells to radiotherapy and cytotoxic drugs that damage DNA, establishing a rationale for using PARP inhibitors as anticancer agents in combination therapy [14, 20]. Clinical proof of concept for synthetic lethality of PARP inhibitors in *BRCA1/2* mutant breast and ovarian tumors has been achieved for olaparib (AstraZeneca) and niraparib (Tesaro) with sustained antitumor activity as monotherapy observed in patients with advanced disease [21, 22, 23, 24]. Olaparib (Lynparza) gained approval from the FDA and the European Medicines Agency for use in certain patients with advanced *BRCA*-mutated ovarian cancer. Additionally, various PARP inhibitors are under evaluation as combinatorial therapy in multiple clinical studies.

TNKS1 and 2 share high sequence similarity with PARP1 within their PARP catalytic domains, however the remainder of the proteins are highly divergent. Specifically, TNKS1 and 2 contain ankyrin repeats for the recognition and binding of substrate proteins and a sterile α-motif (SAM) that mediates protein-protein interaction and self-oligomerization [25, 26]. In contrast, PARP1 comprises a DNA binding domain containing 2 Zn-finger motifs, a domain with a nuclear localization signal, and an auto-modification domain with a BRCT motif [1]. Knockout of genes encoding TNKS1 or 2 individually results in viable and developmentally normal mice, whereas inactivation of both genes is embryonic lethal, analogous to prior findings in PARP1 and 2 knockout mice [27, 28]. Tankyrases have multiple diverse cellular functions including regulation of Wnt/β-catenin signaling, and roles in telomere maintenance, mitosis and glucose uptake [25, 26]. At present, tankyrases are attracting significant attention as emerging therapeutic targets for cancer, principally due to their role in Wnt signaling. Aberrant Wnt/β-catenin signaling has been implicated in the development and progression of multiple cancers. Tankyrase inhibition results in stabilization of axin, a principal constituent of the β-catenin destruction complex, and culminates in antagonism of Wnt signaling [29].

Several PARP inhibitors are currently under evaluation in cancer patients. Phase 3 studies are underway and most are directed toward patients with *BRCA* mutant tumors. In this study, we describe the preclinical profile and characteristics of E7449, a novel and potent inhibitor of PARP1/2 and TNKS1/2. In common with earlier generation PARP1/2 inhibitors e.g. olaparib, niraparib, veliparib (AbbVie), etc., E7449 displays potent antitumor activity in BRCA-deficient *in vivo* models and potentiates the activity of chemotherapy preclinically. Inhibition of TNKS1/2 by E7449 is a significant distinction from traditional inhibitors and the resultant modulation of Wnt/β-catenin signaling may broaden the potential therapeutic applications beyond tumors with deficient DNA repair capacity. Evaluation of E7449 in early clinical studies in cancer patients is underway [30].

## RESULTS

### E7449 inhibits PARP1 and 2 and TNKS1 and 2

**Figure 1:**
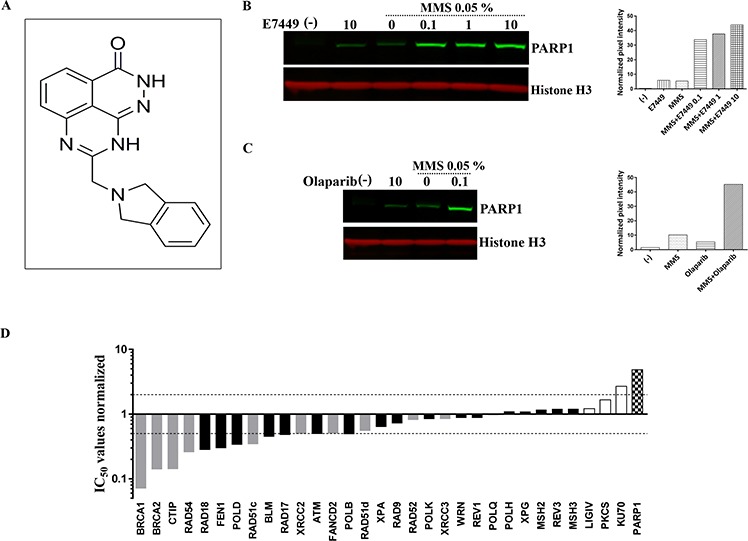
E7449 traps PARP onto DNA and affects DNA repair pathways beyond HR **A.** structure of E7449. **B.** western blot of chromatin-bound fraction from DT40 cells. Cells were treated with various concentrations of E7449 for 30 min or no drug (lanes 1 and 3) in the presence or absence of 0.05% MMS. Chromatin-bound proteins were extracted and subjected to western analysis using antibodies directed against PARP1 or Histone H3, a positive marker for chromatin-bound proteins. Graph represents quantification of PARP1 signal intensity, measured with Image Studio software on the LI-COR Odyssey imager. **C.** western blot of cells treated with olaparib in the presence or absence of 0.05% MMS; graph represents quantitation of PARP1 levels in chromatin-bound fraction. Representative images from 3 independent assays, where E7449 was assayed alongside olaparib. **D.** sensitivity profile of E7449 in a panel of 32 isogenic DNA repair mutant DT40 cell lines. Mean IC_50_ values from at least 3 independent assays were normalized to the IC_50_ value in wild type DT40 cells (3.2 μmol/L). Bars are shaded based on DNA repair function; checkered for PARP1, grey for HR, white for NHEJ, and black for all other DNA repair pathways. Dashed lines represent 2-fold sensitivity or resistance of cell line to E7449 versus the wild type cells.

E7449 is 8-(isoindolin-2-ylmethyl)-2,9-dihydro-3H-pyridazino[3,4,5-de]quinazolin-3-one (Figure 1A, Supplemental Figure 1 for synthesis scheme); an orally bioavailable, brain penetrable, small molecule PARP inhibitor that is not a substrate for P-glycoprotein [33]. Potent inhibition of PARP was observed in a cell free assay (Trevigen) where PARylation of histones was inhibited by E7449 with IC_50_ values of 1.0 and 1.2 nmol/L for PARP1 and 2 respectively (Supplementary Table 1). To examine selectivity of E7449 for PARP1 and 2, a screen of available full length recombinant human PARP enzymes was performed using ^32^P-NAD^+^ as substrate and auto-PARylation as readout [2]. IC_50_ values of ~2.0 and ~1.0 nmol/L were obtained for E7449 inhibition of PARP1 and 2 respectively in this assay (Supplementary Table 1). Significant inhibitory activity was not observed for PARP3 or PARPs 6–16 (PARP9 and 13 lack activity and PARP4 had minimal signal in this study, (data not shown)). In contrast, E7449 inhibited TNKS1 and 2 (PARP5a and 5b) with IC_50_ values of 50–100 nmol/L (Supplementary Figure 2A, Supplementary Table 1). Assay of E7449 with the semi-quantitative TNKS1 histone PARylation assay from Trevigen revealed an average IC_50_ value of 115 nmol/L for E7449 (Supplementary Table 1, Supplementary Figure 2B). In this assay the average IC_50_ value for the selective tankyrase inhibitor XAV939, included as a positive control, was ~10 nmol/L (Supplementary Figure 2B), similar to that previously reported: 11 and 4 nmol/L for TNKS1 and 2 versus 2.194 and 0.114 μmol/L for PARP1 and 2 respectively [29]. In contrast, the selective PARP1/2 inhibitor, olaparib (reported IC_50_ values of 5 and 1 nmol/L for PARP1 and 2 versus 1.5 μmol/L for TNKS1 [34]) did not inhibit tankyrase at the concentrations tested; IC_50_ > 3,000 nmol/L (Supplementary Figure 2B).

### E7449 traps PARP1 onto DNA and affects DNA repair pathways beyond HR

In addition to catalytic inhibition of PARylation, mechanistic studies have recently revealed that PARP inhibitors may act as poisons to trap PARP onto DNA [33–35]. The PARP-DNA complexes likely interfere with DNA replication and thus, contribute to cytotoxicity. In avian B-lymphoblast DT40 cells damaged with the alkylating agent methyl methanesulfonate (MMS) the presence of E7449 resulted in binding of PARP1 to chromatin in a dose responsive manner (Figure 1B). Trapped PARP was also observed upon incubation of cells with olaparib (Figure 1C). Minimal PARP trapping was observed in the absence of MMS or PARP inhibitor (Figures 1B and 1C).

To further evaluate inhibition by E7449 and its selectivity for various DNA repair pathways, a cell proliferation assay was performed in a panel of 32 isogenic DT40 cell lines, in which each line was deficient in a distinct DNA repair gene [31]. In wild type DT40 cells E7449 inhibited cell proliferation in a 2 day assay with an IC_50_ value of 3.2 μmol/L; this value was used for normalization of E7449 IC_50_ values obtained in mutant cells (Figure 1D, see Supplementary Figure 3 for representative IC_50_ curves). Strikingly, DT40 cells lacking PARP expression appeared significantly resistant to treatment with E7449, with a 5 fold increase in IC_50_ versus parental DT40 cells (Figure 1D). A similar observation was made with olaparib inhibition (Supplementary Figure 4): this finding is consistent with the requirement of PARP for drug cytotoxicity and the PARP trapping activity of both inhibitors. Notably, resistance to PARP inhibition was also observed in cells lacking Ku70, a protein required for NEHJ repair of double-strand DNA breaks. As anticipated, lines deficient in components of the HR pathway (BRCA1 and 2, CtIP, Rad54, etc.) were most E7449-sensitive, with IC_50_ values up to 10 fold lower than those observed in wild type DT40 (Figure 1D). In addition, greater than 2 fold sensitization to E7449 was observed in cell lines deficient in checkpoints (ATM and RAD17), ubiquitin ligase for post-replication repair (RAD18), components of BER and nucleotide excision repair (NER) pathways; FEN1, POLD, and POLB, and the RecQ helicase BLM, confirming the extensive inhibitory activity of E7449 in DNA repair, outside the narrow definition of HR (Figure 1D). Overall, the sensitivity profile of E7449 closely resembled that determined for olaparib (Supplementary Figure 4).

### E7449 potentiates antitumor activity of chemotherapies temozolomide and carboplatin

**Figure 2:**
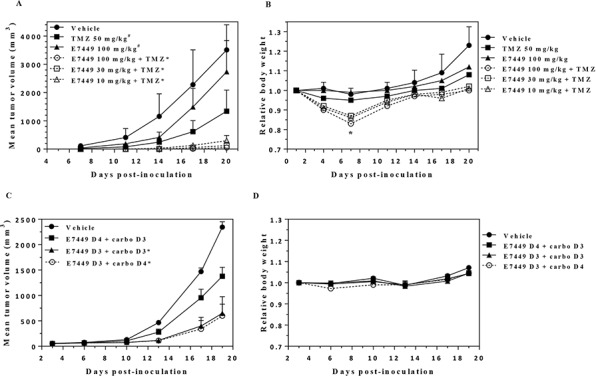
E7449 potentiates antitumor activity of temozolomide and carboplatin **A.** antitumor effect of E7449 in combination with TMZ in B16-F10 mouse melanoma isografts. Data represent the mean ± StdDev. TMZ was administered orally once daily for 5 days. E7449 was orally administered once daily in combination with TMZ for 5 days and alone for an additional 2 days. *P < 0.05 versus TMZ alone on days 14 and 20, ^#^*P* < 0.05 versus vehicle control group on day 14 (one-way ANOVA followed by the Dunnett's multiple comparison test). **B.** relative body weight of animals treated with E7449, TMZ, and E7449 + TMZ combination. Data represent the mean ± SEM. *Body weight loss was observed in all E7449 + TMZ combination treatment groups on day 7. Recovery from body weight loss was observed in all mice upon completion of drug treatment. **C.** antitumor effect of E7449 in combination with carboplatin in a MX-1 human breast cancer orthotopic model. Data represent the mean ± SEM. Carboplatin was administered as a single intravenous dose at 60 mg/kg on day 3 or 4. E7449 was orally administered once daily at 100 mg/kg with administration beginning on either day 3 or day 4. *P < 0.05 versus E7449 D4 + carboplatin D3 on day 19. **D.** relative body weights of animals treated with E7449 + carboplatin combination. No significant body weight loss was observed in any animals over the course of the experiment.

PARP1/2 inhibitors potentiate cell killing effects of DNA damaging chemotherapy through inhibition of DNA damage repair. Consistent and robust chemo-potentiation of the alkylating agent temozolomide (TMZ) has been demonstrated with PARP1/2 inhibition [14, 38, 39]. Potent sensitization of TMZ was established for E7449 in the mouse melanoma B16-F10 isograft model. Growth of subcutaneous tumors was moderately inhibited by single agent treatment with E7449 (100 mg/kg) or TMZ (50 mg/kg), whereas combination of E7449 dosed at 10, 30 and 100 mg/kg with TMZ resulted in significantly enhanced tumor growth inhibition (Figure 2A). The increased antitumor activity was E7449 dose responsive and was significant even at the lowest E7449 dose of 10 mg/kg (Figure 2A). Potentiation of TMZ antitumor therapy was accompanied by increased toxicity as observed by decreased body weight in animals treated with the combination (Figure 2B). No animal lethality occurred but body weight loss was observed in all 3 combination groups. All mice recovered weight once treatment was completed.

Potentiation of platinum agents has also been reported for PARP1/2 inhibitors [14, 40]. In orthotopic MX-1 human breast cancer xenografts, treatment with E7449 enhanced the antitumor activity of carboplatin (Figure 2C). No antitumor activity was observed in MX-1 xenografts following treatment with E7449 alone at 100 mg/kg once daily and administration of a single dose of carboplatin resulted in only modest antitumor activity (Supplementary Figures 5A and 5B). Addition of E7449 resulted in enhanced carboplatin antitumor activity, but only when administered simultaneously with, or prior to carboplatin treatment (Figure 2C). E7449 administration 1 day post-carboplatin treatment resulted in antitumor activity that closely resembled that observed with carboplatin alone. Combination treatment was well tolerated with no signs of toxicity or significant body weight loss observed for any of the treatments (Figure 2D).

### Inhibition of PARP activity and growth of *BRCA* mutant tumors by E7449

**Figure 3:**
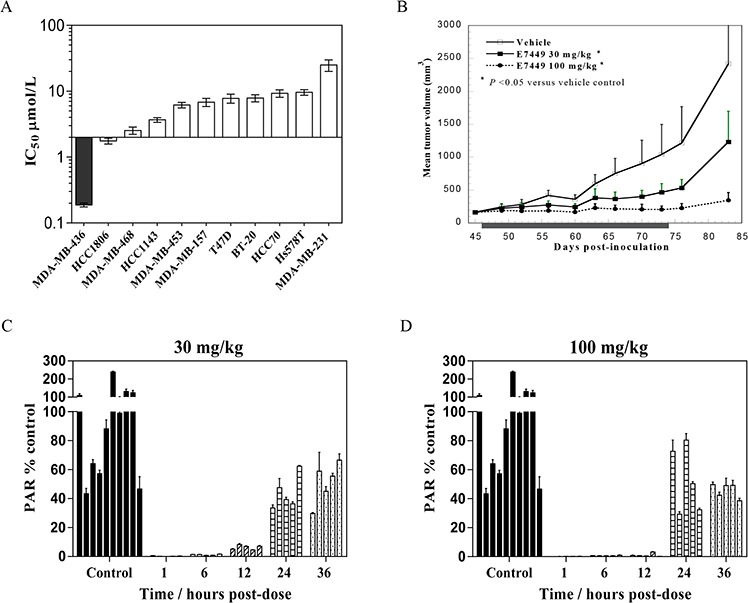
Antitumor activity and PARP inhibition by E7449 in a BRCA mutant xenograft model **A.** E7449 sensitivity profile for inhibition of proliferation in a breast cancer cell line panel. At least 3 independent assays were performed and data represent mean ± SEM. The most E7449-sensitive cell line, MDA-MB-436 (*BRCA1* mutant) is shaded in black. **B.** antitumor effect of E7449 in MDA-MB-436 human breast cancer xenografts. Data represent the mean ± StdDev. E7449 was administered orally once daily for 28 consecutive days as indicated by shaded box. *P < 0.05 versus vehicle control group on day 83 (one-way ANOVA followed by the Dunnett's multiple comparison test). **C.** and **D.** PARP inhibition by E7449 in tumor tissue from MDA-MB-436 human breast cancer xenografts. A single dose of E7449 at 30 mg/kg (Figure 3C) or 100 mg/kg (Figure 3D) was administered to animals bearing MDA-MB-436 tumors. At various timepoints from 1 to 36 hours post-administration, animals were euthanized and tumors harvested. PARP activity in tumor lysate was assessed through determination of PAR levels, normalized by protein concentration. Mean PAR (ng/mg protein) in control animals (vehicle-treated) was set to 100% PARP activity and the inhibition of PARP activity for each time point was calculated by using an average of all control replicates. PAR % of control (mean ± SEM) was calculated from data of 2 experiments assayed in triplicate and each bar on the graph represents % PAR levels in the tumor tissue from an individual mouse.

A compelling body of evidence supports the use of PARP inhibitors in tumors lacking double stranded DNA repair by HR e.g. *BRCA* mutant tumors [reviewed in 14, 15]. To evaluate single agent antitumor activity, E7449 was assessed in a panel of breast cancer cell lines in an 8 day proliferation assay. E7449 sensitive and resistant lines were observed with IC_50_ values ranging from ~0.2 to >10 μmol/L (Figure 3A, see Supplementary Figure 6 for representative IC_50_ curves). MDA-MB-436, a triple negative breast cell line that harbors a *BRCA1* mutation (5396 + 1G > A in splice donor site of exon 20) was the most sensitive cell line identified. In an *in vivo* subcutaneous MDA-MB-436 xenograft model, administration of E7449 at 30 or 100 mg/kg once daily for 28 days resulted in statistically significant antitumor activity (Figure 3B). A dose response was observed, with greater tumor growth inhibition at the higher 100 mg/kg dose. Treatment with E7449 at 30 or 100 mg/kg was well-tolerated without any significant body weight loss or deaths (data not shown). Pharmacodynamic PARP inhibition was assessed in tumors following a single dose of E7449 at 30 or 100 mg/kg (5 mice per group) at various time points post-dosing from 1 to 36 hours (Figure 3C and 3D). Considerable variability in PAR levels was observed in the control (vehicle treated) group of 10 mice, as previously reported [41]. Treatment with E7449 at 100 mg/kg resulted in significant PARP inhibition that was sustained for at least 12 hours and recovered to basal levels within 24 hours (Figure 3D). At the 30 mg/kg dose significant PARP inhibition was also observed but the effect was less sustained and partial rebound was observed within 12 hours (Figure 3C).

### Pharmacodynamic PARP inhibition by E7449 in tumors

**Figure 4:**
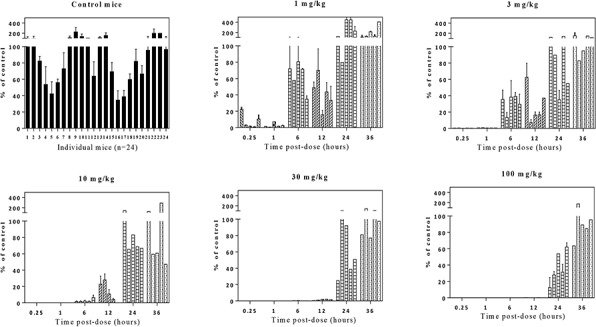
Dose-dependent PARP inhibition by E7449 in tumor tissue from NCI-H460 human lung cancer xenografts Mice were administered a single E7449 dose from 1 to 100 mg/kg or vehicle (control group). Mice were euthanized and tumors harvested for PAR analysis by ELISA at various time points from 15 min to 36 hours post-treatment. Normalization was performed as outlined in MDA-MB-436 study. PAR % of control (mean ± SEM) was calculated from data of 2 experiments assayed in triplicate. Each bar in graph represents % PAR level in the tumor tissue from an individual mouse.

To further interrogate E7449 pharmacodynamic PARP inhibition a study was conducted in the NCI-H460 lung cancer xenograft model. No antitumor activity was recorded for E7449 in this model which was selected for its rapid and consistent tumor growth. Mice were administered a single E7449 dose from 1 to 100 mg/kg and tumors were harvested for PAR analysis by ELISA at various time points from 0.25 to 36 hours post-treatment. As in the previous study, significant variability in tumor PAR levels of vehicle-treated mice was noted (Figure 4, control mice panel). E7449 treatment resulted in significant PARP inhibition at early time points (0.25 and 1 hour) even at the lowest administered dose of 1 mg/kg (Figure 4). PARP activity levels were recovered at least partially to basal levels within 6 hours post-dose of E7449 at 1 and 3 mg/kg. Increasing E7449 dose resulted in a more sustained duration of PARP inhibition. At the highest E7449 dose of 100 mg/kg complete inhibition was observed up to 12 hours and the rebound in activity delayed until 24 hours post treatment (Figure 4).

### E7449 inhibits Wnt signaling *in vitro*

**Figure 5:**
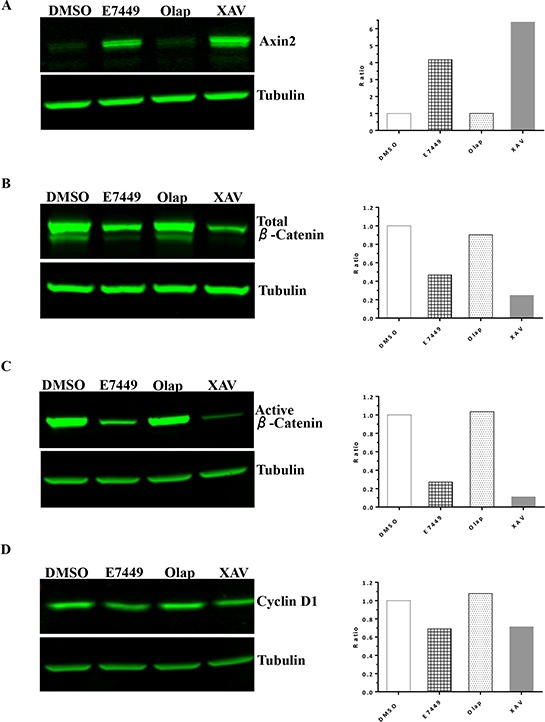
E7449 inhibits Wnt signaling *in vitro*: effects of E7449 treatment on Wnt proteins in SW480 cells by western blot analysis Following 24 h incubation of cells with indicated compounds at 10 μmol/L, cell lysates were subjected to electrophoresis and western blot, then probed with antibodies targeting various Wnt/β-catenin pathway proteins: **A.** axin2; **B.** total β-catenin; **C.** active (non-phosphorylated) β-catenin; **D.** cyclin D1. Tubulin was used as a sample loading control and fluorescence intensity of bands was measured using Image Studio software on the LI-COR Odyssey imager. Ratio of analyte to tubulin was plotted (A–D, right hand panels) and each is a representative of several independent experiments.

Tankyrase inhibition antagonizes Wnt/β-catenin signaling through axin stabilization, which results in enhanced activity of the destruction complex and ultimately in proteolysis of β-catenin [29]. Studies were performed to determine if E7449-mediated tankyrase inhibition could affect Wnt signaling in human colon cancer SW480 (Wnt active, *APC* mutant) cells *in vitro*. A significant increase in the level of axin2 protein was observed in cells treated with E7449 (Figure 5A). Increased axin2 was also observed following treatment with the selective tankyrase inhibitor XAV939, whereas axin2 remained at basal levels following treatment with the selective PARP1/2 inhibitor olaparib (Figure 5A). Stabilization of axin2 as well as tankyrase proteins appeared both E7449-dose and time responsive (Supplementary Figures 7A and 7B). Concomitant with the increase in axin2, E7449 treatment reduced levels of active (non-phosphorylated) and total β-catenin by ~70 and 50% respectively, versus ~90 and 75% for the more potent XAV939 (Figures 5B and 5C). In contrast, β-catenin protein levels remained unchanged in olaparib-treated cells (Figures 5B and 5C). A modest decrease in Cyclin D1 protein levels was observed following treatment with E7449 or XAV939, while protein levels were again unaffected by olaparib treatment (Figure 5D). Together these data suggest that alterations in Wnt/β-catenin signaling proteins, mediated by E7449 and XAV939, are the consequence of tankyrase inhibition and accordingly were not observed with olaparib, which only weakly inhibits tankyrase.

In DLD-1 cells (Wnt active, *APC* mutant) treated with E7449, similar if less potent effects were observed on axin2 and cyclin D1; analogous to effects induced by XAV939, but not olaparib (Supplementary Figures 7C and 7D). A robust effect on levels of β-catenin was not observed by western blot for E7449 or XAV939 in this cell line. In the Wnt inactive human colon cancer RKO cell line, axin2 and β-catenin were not detected (data not shown).

**Figure 6:**
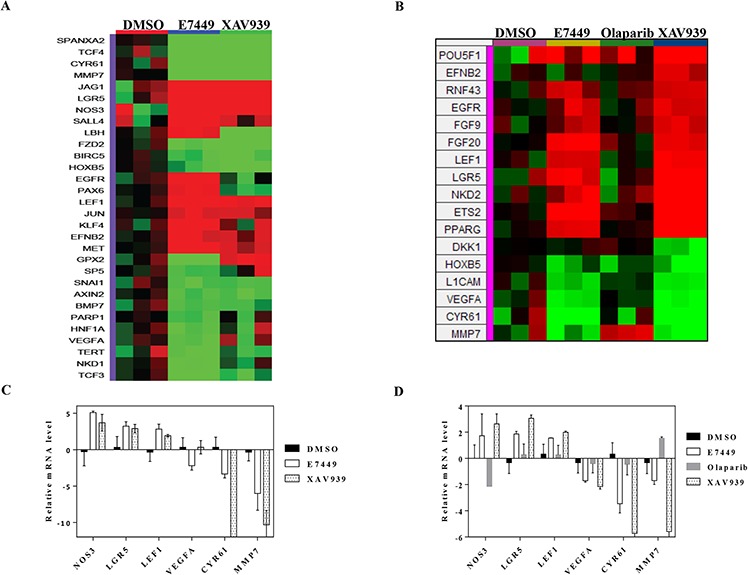
E7449 inhibits Wnt signaling *in vitro*: effects of E7449 treatment on expression of Wnt-related genes in SW480 cells **A.** following 72 h incubation of SW480 cells with E7449 or XAV939 at 30 μmol/L or DMSO (control), RNA was harvested and gene expression profiling performed using a custom designed TLDA; genes listed in Supplementary Table 2. The heat map represents 30 Wnt-related genes whose expression was altered following E7449 exposure (red: increased, green: decreased). Genes with a relative fold change of ≥ 1.5 (*P* > 0.05, student's *t*-test) versus DMSO control were subjected to hierarchical clustering (Manhattan distance) and complete linkage plotting to generate the heat map. **B.** Gene expression profiling in SW480 cells treated with E7449, XAV939 or olaparib at 3 μmol/L or DMSO for 72 h. Heat map generated as above represents 17 Wnt-related genes whose expression was significantly altered by any of the 3 compound treatments. **C.** and **D.** 6 E7449-responsive genes common to both 3 and 30 μmol/L study respectively; data represent mean ± SEM. In general, XAV939 treatment altered gene expression in the same direction as E7449, whereas olaparib treatment had no effect or altered expression in the opposite direction (D).

Gene expression profiling was performed to measure the effect of E7449 treatment on expression of genes involved in Wnt signaling. Expression was measured by quantitative PCR using a custom-designed array following E7449 treatment of SW480 cells. Significantly altered expression of 30 Wnt-related genes was observed following E7449 treatment. Overall, the gene expression profile revealed by E7449 treatment closely resembled that obtained with XAV939 (Figure 6A). E7449-treated DLD-1 cells also underwent significantly altered expression of 40 Wnt-related genes and again, the expression heat map closely resembled that of XAV939 treated-cells (Supplementary Figure 8). Approximately 45% of genes altered upon E7449 treatment were common to both cell lines. PARP inhibitors are known to act as regulators of transcription factors [42]; therefore a study was conducted to confirm that gene changes observed were the result of tankyrase inhibition by E7449 and not PARP1/2 inhibition. SW480 cells were treated with E7449, XAV939 or olaparib (at 3 μmol/L where olaparib is not expected to inhibit tankyrases, as compared with 30 μmol/L in the previous study), and gene expression changes were measured using the array described above. Expression of 17 Wnt-related genes was significantly altered by at least one of the 3 compound treatments (Figure 6B). Heat map expression profiles for E7449 and XAV939 were again highly comparable and differed considerably from that of olaparib, whose profile more closely resembled that of DMSO. Six E7449-responsive genes common to each study (*NOS3, LGR5* and *LEF1* up-regulated and *NKD1, CYR61* and *MMP7*, down-regulated) are represented in Figure 6C and 6D. XAV939 treatment also altered expression of these 6 genes in the same direction, although differences in the scale of change were observed (Figure 6C and 6D). In contrast, olaparib treatment had no effect or altered expression in the opposite direction (*NOS3, MMP7*), (Figure 6D) suggesting that the E7449-mediated gene expression changes are the consequence of tankyrase inhibition.

### E7449 inhibits Wnt signaling *in vivo*

**Figure 7:**
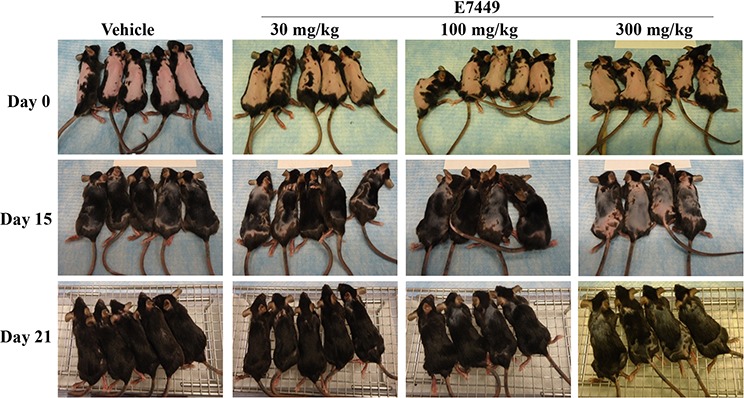
E7449 dose-responsively inhibits re-growth of hair, a Wnt-mediated pathway, in mice C57BL/6 female mice were depilated using Nair™ and E7449 treatment initiated the following day. E7449 was administered at 30, 100 or 300 mg/kg once daily for 12 days. Re-growth of hair was monitored and recorded by photography for comparison to vehicle-treated mice.

Wnt signaling is necessary for hair follicle development and cycling [43–45]. Re-growth of hair in mice was investigated to determine if tankyrase inhibition by E7449 could impact Wnt signaling *in vivo*. Following hair removal, C57BL/6 mice were treated with E7449 administered once daily at 30, 100 or 300 mg/kg. Within 2 weeks, notable re-growth of hair was observed in vehicle-treated mice with only a few small bald patches remaining; full hair re-growth was observed by Day 21 (Figure 7). In contrast, hair re-growth was significantly delayed in mice treated with E7449. A dose response effect was observed and bald patches remained at Day 21 in mice treated with the higher E7449 doses (Figure 7). These data suggest inhibition of Wnt signaling *in vivo* mediated by E7449, likely through inhibition of tankyrase activity.

### E7449 combined with MEK inhibitor inhibits tumor growth in a Wnt model

**Figure 8:**
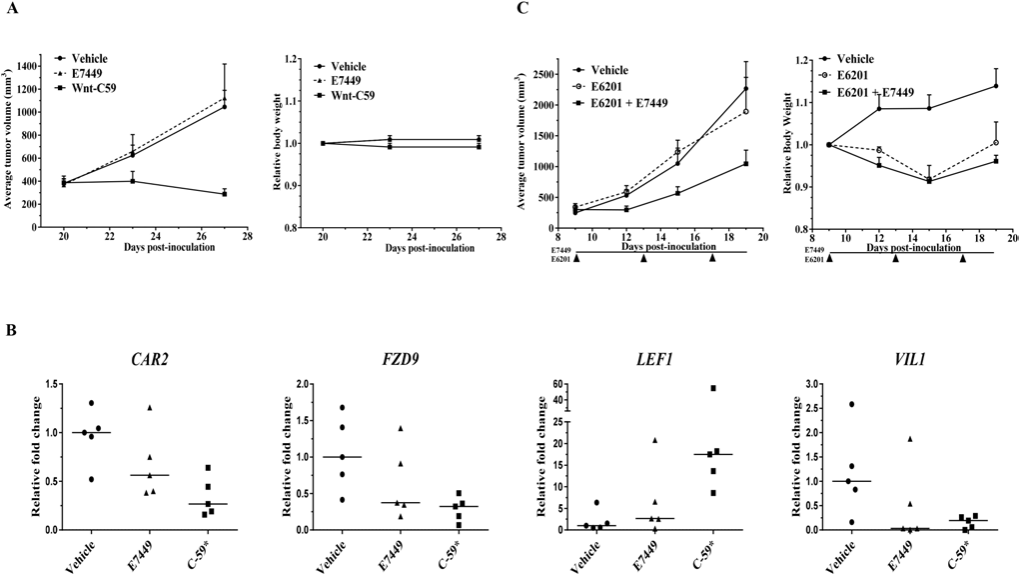
Antitumor effect of E7449 in combination with MEK inhibitor in Wnt-dependent model **A.** effect of E7449 on tumor growth in Wnt1 subcutaneous model. No antitumor effect was observed following administration of E7449 once daily at 100 mg/kg. Wnt-C59 porcupine inhibitor dosed once daily at 10 mg/kg significantly inhibited tumor growth (left panel). Both drugs were well tolerated with minimal toxicity as measured by body weight loss (right panel). **B.** Relative fold change in expression of *CAR2, FZD9, LEF1,* and *VIL1* in tumors harvested following 7 days of drug treatment. Analysis was performed using a mouse-specific TLDA (genes listed in Supplementary Table 3) and data for individual tumors are plotted (5 mice per group), with lines representing median. * *P* > 0.05, student's *t*-test with fold change of ≥ 2. **C.** synergistic antitumor activity with combination of E7449 and MEK inhibitor E6201 in Wnt1 model. E7449 was administered orally once daily and E6201 was administered intravenously Q4Dx3. No antitumor activity was observed with single agent E6201; combination with E7449 resulted in synergistic inhibition of tumor growth (left panel). E6201 treatment resulted in decreased body weight (<10%) which recovered post-treatment. Addition of E7449 to E6201 did not impact body weight loss (right panel).

In a Wnt1 subcutaneous model (mammary tumors initially isolated from Wnt1 (int-1) transgenic mice [32]), single agent E7449 treatment (100 mg/kg) did not inhibit tumor growth whereas significant antitumor activity was observed following administration of the porcupine inhibitor Wnt-C59, a potent Wnt signaling antagonist (Figure 8A). Gene expression analysis in tumors from Wnt-C59-treated mice revealed significant alteration of several Wnt-related genes including *CAR2, FZD9, LEF1,* and *VIL1* (Figure 8B). Expression of these genes was also altered in tumors from E7449-treated mice, albeit to a lesser extent (Figure 8B). Tankyrase inhibition of Wnt signaling by itself may prove insufficient to achieve tumor shrinkage; therefore, activity of E7449 was re-assessed in combination with the MEK inhibitor E6201. Antitumor activity was enhanced in the combination versus either single agent alone (Figure 8C, left panel). The addition of E7449 to E6201 had minimal effect on toxicity as measured by body weight loss (Figure 8C, right panel).

## DISCUSSION

This report provides the first characterization of a dual PARP1/2 and TNKS1/2 inhibitor. E7449 is a novel, potent inhibitor of the DNA repair proteins PARP1 and 2; it traps PARP onto DNA to augment cytotoxicity and, comparable to earlier PARP inhibitors it exhibits selectivity for tumor cells that are deficient in HR repair and other DNA repair pathway proteins. Single agent antitumor activity was observed for E7449 in a *BRCA* mutant xenograft model, while in combination E7449 potentiated the DNA damaging effects of chemotherapy *in vivo*. Additionally, and in contrast to described clinical PARP inhibitors, E7449 inhibits the PARylation activity of TNKS1/2 at a clinically relevant dose. Tankyrase is a key regulator of Wnt/β-catenin signaling, a pathway known to promote tumorigenesis and a target for the development of cancer therapeutics. In cells with active Wnt signaling, E7449 treatment altered expression of Wnt pathway proteins and Wnt-related genes, and administration to mice prevented re-growth of hair, a Wnt-dependent pathway. Pharmacodynamic effects on Wnt signaling were observed in the Wnt1 model, and co-administration of E7449 with a MEK inhibitor, resulted in a synergistic antitumor effect, with no significant toxicity. Dual inhibition of TNKS1/2 and PARP1/2 distinguishes E7449 from existing PARP inhibitors and may lead to a distinct spectrum of clinical opportunity; E7449 is currently in early phase clinical development [30].

E7449 inhibits PARP1 and 2 with a potency of 1.0 and 1.2 nmol/L; values in a similar range to those reported for olaparib, niraparib and talazoparib (BioMarin), with the caveat that different assays were used for each inhibitor [34, 46, 47]. PARP trapping was demonstrated for E7449 in DT40 cells in the presence of the alkylating agent MMS. A dose responsive effect was observed, however the increase in trapped PARP appeared minimal alongside the 100 fold increase in E7449 concentration (Figure 1B), perhaps because PARP trapping was close to maximal at the lowest E7449 treatment (0.1 μmol/L), or reflecting the limit of sensitivity of the assay. The amount of PARP detected in chromatin complexes was almost identical for E7449 and olaparib. Beyond the similar potency of catalytic inhibition and PARP trapping by E7449, the dose of E7449 necessary for sustained PARP inhibition in tumors and single agent antitumor activity closely resembles that described for the traditional PARP inhibitors, olaparib, niraparib, etc. rather than the lower dosed talazoparib [34, 46, 47].

Data generated in the DT40 cell line panel where PARP deficient cells were significantly more resistant to E7449 than wild type cells demonstrate the requirement of PARP for E7449 activity and also confirm E7449-mediated PARP trapping to enhance cytotoxicity. Resistance to E7449 was also observed in cell lines deficient in NHEJ repair (*KU70*, *PKCS*, and *LIGlV*) as previously reported for other PARP inhibitors [37]. Predictably, cell lines that lacked HR pathway proteins were more sensitive to E7449, and an increase in susceptibility to E7449 was also observed in cell lines with deficiency in checkpoint proteins and well as proteins from several DNA repair pathways, including BER and NER. The profile of E7449 in the panel is indistinguishable to that of olaparib, again demonstrating that the PARP1/2 inhibition properties of E7449 closely correlate to those of traditional PARP inhibitors.

While E7449 closely parallels previously described inhibitors in terms of PARP inhibition and preclinical properties, significant divergence arises with respect to effects on tankyrase activity. E7449 inhibits TNKS1/2 with IC_50_ values from 50 to 120 nmol/L, a dose considered clinically relevant [30]. An IC_50_ value of > 3 μmol/L was determined for olaparib for TNKS1 inhibition using the Trevigen assay. Reported IC_50_ values for tankyrase inhibition by olaparib, niraparib, and veliparib (various assays) are at least 5–20 fold higher than that observed for E7449 (1.5 μmol/L, 0.6 μmol/L, and 15 μmol/L, respectively [34, 46, 25]), and reflect drug concentrations not likely to be achievable clinically. No data have been reported for tankyrase inhibition by rucaparib or talazoparib, although rucaparib binding to the catalytic domain of multiple PARP family members including tankyrases was described by Wahlberg et al [48]; no significant binding of TNKS1/2 was reported for olaparib or veliparib in their study.

While tankyrases perform multiple and varied cellular tasks including maintenance of telomeres, roles in mitosis and glucose uptake, the burgeoning interest in targeting tankyrase for development of anticancer therapeutics is primarily directed to their role in the regulation of Wnt/β-catenin signaling [25, 26]. The β-catenin destruction complex (composed of APC, axin (limiting component), and GSK3β) regulates proteolysis of the transcription factor β-catenin through phosphorylation and is inactivated during active Wnt signaling; this leads to accumulation of non-phosphorylated β-catenin that translocates to the nucleus and transcribes multiple target genes, including cyclin D1. PARylation of axin by tankyrase is necessary for its subsequent ubiquitination by RNF146 and proteasome degradation; inhibition of tankyrase by XAV939 results in axin and tankyrase stabilization, enhanced activity of the destruction complex and ultimately in inhibition of β-catenin-mediated transcription [25, 29, 49]. Wnt/β-catenin signaling perturbation was achieved with E7449 treatment in Wnt-active colon cancer cells and the profiles generated both by western blot and in gene expression studies appeared very similar to that of the selective tankyrase inhibitor XAV939. Importantly and distinctly, treatment with olaparib which lacks potent tankyrase inhibition had minimal impact on Wnt signaling proteins in these *in vitro* studies, implying that effects were not PARP1/2-sensitive and were more likely the result of tankyrase inhibition. Additionally, E7449 treatment prevented re-growth of hair in mice, a process that is Wnt signaling dependent [43–45]. We postulate that E7449 reduces Wnt/β-catenin signaling by inhibiting tankyrase, thus preventing PARylation-dependent axin degradation, and thereby promoting β-catenin destabilization.

Tankyrase is currently the most highly validated druggable target in the Wnt/β-catenin pathway; inhibitors have been shown to reduce signaling and extensive discovery efforts have resulted in the identification of multiple tankyrase inhibitors [reviewed in 25, 26]. Of these, only G007-LK was reported to inhibit tumor growth as a single agent in certain models [50], while the majority of tankyrase inhibitors lack antitumor activity *in vivo*. Similarly, E7449 treatment resulted in pharmacodynamic effects on Wnt-target genes *in vivo* but these changes in gene expression appeared insufficient to mediate an antitumor effect in the Wnt1 model as a single agent. G007-LK has a very narrow therapeutic window because of intestinal toxicity, an on-target tankyrase inhibition effect [50]. Dose limiting intestinal toxicity that produces significant adverse effects is commonly observed upon inhibition of Wnt/β-catenin signaling [51, 52]. Importantly, no gastrointestinal toxicity was observed in preclinical studies with E7449 despite evidence of Wnt-target perturbation. Moreover, E7449 has been generally well tolerated in cancer patients treated to date, with fatigue (a PARP inhibitor class effect) rather than intestinal toxicity identified as the dose-limiting toxicity in a small phase 1 study [30].

Wnt/β-catenin signaling has been identified as a potential mediator of resistance to MEK inhibition and strong synergy has been observed for the combination of MEK and tankyrase inhibition in *KRAS*-mutant cancer cells [50, 53, 54]. Consistent with these findings, when E7449 was combined with the MEK inhibitor, E6201, synergistic antitumor activity was observed in the Wnt1 model. E7449 also significantly potentiated the antitumor effects of temozolomide and carboplatin with tolerable toxicity, most likely through inhibition of DNA repair activity of PARP1/2. In addition to a wide range of chemotherapeutic agents, PARP inhibitors are increasingly under clinical investigation in combination with targeted therapies including inhibitors of PI3K, bortezomib, etc. Co-inhibition of TNKS1/2 by E7449 potentially increases the range and number of possible, rationally targeted combinations for this therapy. For example, a critical role for tankyrase and Wnt/β-catenin signaling was identified for maintenance of lung cancer cells during EGFR inhibition and subsequent inhibition of tankyrase significantly enhanced the antitumor activity of EGFR inhibitors in NSCLC cells [55]. Testing additional targeted therapies with E7449 may reveal novel combinations and indications for further development.

In this study we illustrate the unique properties of E7449, a multi-targeted drug. We provide evidence for meaningful inhibition of the DNA repair PARPs, PARP1/2, in addition to TNKS1/2, key components of Wnt signaling. Inhibition of multiple anticancer targets offers the potential for enhanced efficacy and expanded indications or combination partners, versus a single target drug. Multi-target agents are common in drug discovery and promiscuous multi-kinase inhibitors have proved therapeutically effective anticancer drugs; using this as an example, we propose that E7449 may possess increased or broader therapeutic effectiveness through its dual PARP/TNKS inhibition.

## MATERIALS AND METHODS

### Reagents

Synthesis of E7449 (C_18_H_15_N_5_O; IUPAC 8-(isoindolin-2-ylmethyl)-2,9-dihydro-3H-pyridazino[3,4,5-de]quinazolin-3-one is described in the supplementary methods and structures of intermediates are provided in Supplementary Figure 1. E7449 and E6201 (Eisai Inc.) stock solutions were prepared in dimethyl sulfoxide (DMSO), aliquoted and stored at −20°C. Olaparib and XAV939 were obtained from Selleck Chemicals. Temozolomide (TMZ) was obtained from LKT Laboratories (Cat# T1849).

A panel of 32 isogenic DT40 cell lines [31], in which each line was deficient in a distinct DNA repair gene was maintained and assays were performed in the laboratory of Dr. Jun Nakamura, UNC. MX-1 cells were obtained from National Cancer Institute (Bethesda, MD). All other cell lines were obtained from American Type Culture Collection (ATCC) and maintained according to their instructions. For *in vivo* studies, cells were used within a short time of receipt from ATCC or cell line authenticity was verified by STR typing.

### PARP and TNKS enzyme assays: catalytic inhibition and PARP trapping

The ability of E7449 to inhibit the activity of human recombinant PARP1, mouse recombinant PARP2 or human recombinant TNKS1 was determined using chemiluminescent PARP or tankyrase assay kits from Trevigen, following the manufacturer's instructions. IC_50_ values were determined by non-linear regression using the GraphPad Prism 5 software version 5.02 (Lake Forest, CA).

DT40 cells [31] were treated with PARP inhibitor (0.1–10 μmol/L) in combination with 0.05% Methyl methanesulfonate (MMS, Sigma-Aldrich (Cat# 129925). Cells were pretreated for 5 min with MMS prior to adding PARP inhibitor for a further 30 min incubation at 37°C with gentle shaking. Following treatment, cells were lysed using the subcellular protein fractionation kit for cells (Thermo Scientific, Cat# 78840) to obtain the chromatin-bound fraction. Chromatin-bound proteins were subjected to western blot analysis with antibodies against PARP1 (Cell signaling Technology (CST), Cat# 9532), and histone H3 as control (CST Cat# 3638). Following binding of appropriate secondary antibodies labeled with IRDye^®^ (LI-COR), image analysis and quantitation of blots was performed with Image Studio software for the LI-COR Odyssey system (version 2.1.10).

GFP-PARPs were expressed in 293F cells and the auto-PARylation activity of each PARP was assessed in the presence or absence of E7449 at various concentrations as in the previously described assay [2]. Briefly, 24 to 48 h after transfection, cells were washed 3x in ice-cold PBS and lysed for 20 min on ice in cell lysis buffer (CLB: 50 mM HEPES, pH 7.4, 150 mM NaCl, 1 mM MgCl_2_, 1 mM EGTA, 1 mM DTT, 1% TritonX-100, 1 μg/mL leupeptin, aprotinin, pepstatin, PMSF). Lysates were subject to ultracentrifugation at 100,000 *g* for 30 min. Cleared lysates were incubated for 1 h at 4°C with anti-GFP antibody (3E6, Life Technologies) and pre-bound protein A magnetic beads (Millipore). Beads were then washed 1 × 5 min in CLB, followed by 3 × 10 min washes in CLB containing 1 M NaCl, and 1 × 5 min wash in PARP reaction buffer (PRB; 50 mM Tris, pH 7.5, 50 mM NaCl, 0.5 mM DTT, 0.1% TritonX-100, 1 μg/mL leupeptin, aprotinin, pepstatin). NAD^+^ incorporation reactions were performed in PRB containing 10 μmol/L NAD^+^ supplemented with ^32^P-NAD^+^ at 1:20 ratio for 30 min at 25°C. For PARPs with low incorporation signals (PARP4, 5a and 16), NAD^+^ incorporation was performed at 1:5 ratio for 1 h at 25°C. Beads were then re-suspended in Laemmli sample buffer, heated to 65°C for 10 min, the beads removed using a magnet, and the supernatant spotted onto Whatman paper. Samples were analyzed via phosphorimaging.

### Cell lines and proliferation assay

Proliferation assays were performed in a panel of 32 isogenic DT40 cell lines, in which each line was deficient in a distinct DNA repair gene [31]. Cells were seeded and incubated with test compound at various concentrations for 2–3 days (~ 8 cell cycles). Cell growth was assessed using XTT (ATCC^®^ Cat# 30–1011K™) and IC_50_ values were calculated using the GraphPad Prism 5 software version 5.02 (Lake Forest, CA). Each experiment was conducted in duplicate and a minimum of 3 separate experiments were performed. Human breast cancer cell lines, HCC1143, HCC70, HCC1806, MDA-MB-436, T47D, MDA-MB-157, MDA-MB-231, MDA-MB-468, MDA-MB-453, BT-20 and Hs578T were obtained from ATCC. For cell line panel assays, cells were maintained and assayed in RPMI 1640 or DMEM medium containing 10% FBS. For proliferation assays cells were plated at low density in 96 well plates. E7449 was added at various concentrations and plates incubated for a total of 8 days; compound and medium were replenished on day 4. Cell growth was assessed using the CellTiter-Glo^®^ cell viability assay (Promega, Cat# G7573). Each experiment was conducted in duplicate and a minimum of 3 separate experiments were performed.

### Western blot and Gene expression

SW480, DLD-1 (Wnt-dependent) or RKO (Wnt-independent) cells were plated in 6-well plates and incubated overnight. Cells were treated with E7449, olaparib or XAV939 and incubated for various time periods prior to processing by SDS PAGE and western blot analysis. Blots were probed with antibodies to axin 2 (CST Cat# 2151), total β-catenin (CST Cat# 8480), non-phospho (active) β-catenin (CST Cat# 8814), cyclin D1 (CST Cat# 2926, Cat#2978), tankyrase (Abcam Cat# ab13587: this antibody does not discriminate between tankyrase 1 and 2), α-tubulin (Abcam Cat# ab56676), and β-actin (Abcam Cat# ab3280). Following binding of appropriate secondary antibodies labeled with IRDye^®^ (LI-COR), image analysis was performed with Image Studio software for the LI-COR Odyssey system (version 2.1.10).

Gene expression profiling was performed using a custom designed TaqMan^®^ low density array (TLDA). Total RNA was isolated from cell lines using RNeasy Mini Kit (Qiagen) and from xenograft tumor FFPE tissue using RecoverAll Total Nucleic Acid Isolation (Ambion). Human and mouse, real-time qPCR TLDAs (384-well micro fluidic card) were designed with genes selected based on their involvement in Wnt signalling (see supplementary Tables 2 and 3 for lists of genes on TLDAs). cDNA was generated from total RNA using High Capacity cDNA Reverse Transcription kit (Life Technologies) or SuperScript^®^ VILO™ Master Mix (Life Technologies). 100 ng cDNA was combined with TaqMan^®^ Gene Expression Master Mix (Life Technologies) for the cell lines and TaqMan^®^ Advanced FAST Master Mix (Life Technologies) for the xenograft tissue for each port (in duplicate). TLDAs were run based on recommended cycling times for each master mix. Ct values were calculated and imported into GeneData Analyst for further analysis. To calculate the ΔCts, gene expression was normalized to several housekeeping genes. Relative fold change was calculated by comparison to the DMSO or vehicle-treated controls for each sample. A student's *t*-test was used to calculate *P*-values and hierarchical clustering used Manhattan distance and complete linkage plotting of selected significant genes to generate heat maps.

### *In vivo* efficacy studies

All studies were performed according to IACUC approved protocols. The general health of mice was monitored daily. Tumor volume was determined by caliper measurements (mm), using the formula (l x w^2^)/2 = mm^3^, where l and w refer to the larger and smaller perpendicular dimensions collected at each measurement. Tumor dimensions were recorded twice per week starting when tumors reached an approximate size of 100 to 150 mm^3^. Body weights were recorded twice per week and relative body weight was calculated as follows: Relative body weight = (body weight on day of measurement/body weight on first day of treatment).

TMZ combination in B16-F10 isograft model: female C57BL/6 mice were inoculated subcutaneously with B16-F10 cells (2 × 10^5^). Following randomization by body weight, drug treatment was initiated 1 day post-inoculation. Both E7449 and TMZ were formulated in 0.5% methyl cellulose and orally administrated once per day. TMZ was administered daily on days 1 to 5 at 50 mg/kg as a single agent or in combination. E7449 was administered daily on days 1 to 7 at doses of 10, 30 and 100 mg/kg in combination with TMZ and at a dose of 100 mg/kg as a single agent. The control group was treated with vehicle (0.5% methyl cellulose in water). E7449 or vehicle was administered first and when dosing of all animals was complete TMZ was administered to animals receiving the combination.

Carboplatin combination in MX-1 orthotopic xenografts: female nude mice were implanted with MX-1 cells (0.5 × 10^6^) in the thoracic mammary fat pad. Treatment started on day 3 when the average tumor size was approximately 50 mm^3^. Carboplatin was administered as a single intravenous dose at 60 mg/kg on day 3 or 4. E7449 was administered orally once daily at 100 mg/kg with administration beginning on either day 3 or day 4.

E7449 as a single agent in MDA-MB-436 xenografts: female SCID mice were inoculated subcutaneously with MDA-MB-436 cells (1 × 10^7^). E7449 treatment was initiated on day 46 post-inoculation and continued once daily for 28 days at doses of 30 or 100 mg/kg.

E6201 and E7449 combination in Wnt1 model: Mammary tumors were initially isolated from Wnt1 (int-1) transgenic mice [32] and the model maintained through serial passage of tumor pieces. Tumor pieces cut to approximately 2 mm^3^ in size were injected subcutaneously in female nude mice. Treatment was initiated approximately 9–10 days post-implantation of mice. E7449 was administered once daily at 100 mg/kg, alone or in combination with E6201 (MEK inhibitor), administered intravenously every 4 days 3 times at 40 mg/kg. The porcupine inhibitor Wnt-C59 (BioVision Cat# 2063) which inhibits Wnt signaling was included as a positive control and dosed orally once daily at 10 mg/kg (formulated in 0.5% methyl cellulose with 0.1% Tween 80).

Seven week old C57BL/6 female mice were subjected to depilation using Nair™ to examine any effect of E7449 on re-growth of hair. Drug treatment was initiated the next day and E7449 was dosed at 30, 100 or 300 mg/kg orally once daily for 12 days. Re-growth of hair was monitored and recorded by photography.

### *In vivo* PARP inhibition

Inhibition of PARP activity was evaluated in tumor lysates from MDA-MB-436 xenograft mice following administration of a single dose of E7449 at 30 or 100 mg/kg. Control group (10 mice) were treated with vehicle (0.5% methyl cellulose in water). Mice were euthanized and tumors were harvested at 1, 6, 12, 24 and 36 hours post treatment (5 mice per treatment per time point). Individual tumors were removed and flash frozen in liquid nitrogen for PAR analysis. PARP inhibition was also evaluated in tumors from NCI-H460 human lung cancer subcutaneous xenografts. Control group animals (24 mice) were treated with vehicle (0.5% methyl cellulose). E7449 was administered in a single dose at 1, 3, 10, 30 and 100 mg/kg. Mice were euthanized and tumors were harvested at 0.25, 1, 6, 12, 24 and 36 hours post-treatment. PAR levels were determined by ELISA using the procedure provided by Trevigen and comparing to a PAR standard curve. Protein concentration of tumor lysates was determined using the BCA assay (Thermo Scientific). Results were converted from pg PAR/assay well to ng PAR/mg protein. Data was pooled from multiple experiments. The percent of control for each time point was calculated by using an average of all control replicates from that experiment.

## SUPPLEMENTARY METHODS


