# Supplementary material for: E7449: A dual inhibitor of PARP1/2 and tankyrase1/2 inhibits growth of DNA repair deficient tumors and antagonizes Wnt signaling
Source: Oncotarget. 2015 Oct 20;6(38):41307–23. doi: 10.18632/oncotarget.5846 (PMC4747407; doi:10.18632/oncotarget.5846)
Supplement: Supplementary file 1 [file oncotarget-06-41307-s001.pdf]

SUPPLEMENTARY METHODS

SYNTHESIS OF E7449

To a solution of 4N HCl in dioxane (4.8 L, 4.0 equiv) was added chloroacetonitrile (1.2 L, 4.0 equiv) dropwise at such a rate as to maintain the internal temperature at NMT 30°C. The resulting solution was allowed to stir for 30 minutes at room temperature, after which a solution of compound **1** (1.0 kg, 1.0 eq) in dioxane (500 mL) was added dropwise at such a rate as to maintain internal temperature at NMT 60°C. The mixture was heated to 90°C until HPLC analysis indicated complete reaction (typically 3 hours), then cooled at RT for 30 mins. The mixture was filtered to afford methyl 2-(chloromethyl)-4-oxo-1,4-dihydroquinazoline-5-carboxylate (**2**, 1.32 kg, 95% yield).

To a solution of compound **2** (1 kg, 1 equiv) in DMF (7 L) was added diisopropylethylamine (Hunig's base, 0.6 L, 1 equiv) dropwise, maintaining internal temperature ≤ 30°C. Isoindoline hydrochloride (0.7 kg, 1.3 equiv) was added followed by additional diisopropylethylamine (Hunig's base, 1.5 L, 2.6 equiv) dropwise, maintaining internal temperature at ≤ 25°C. The mixture was allowed to stir for 16 h until HPLC analysis indicated complete reaction. Under a flow of nitrogen, water (7 L) was added at such as rate as to maintain internal temperature ≤ 35°C. The solution pH was carefully adjusted to pH = 6.0 using 6N HCl (ca. 0.4 L). The mixture was allowed to stir at room temperature for 30 mins, then filtered. The solid was washed 2 times with 2:1 water/DMF (3L), then once with water (3 L), then once with MTBE (1 L). The solid was dried under vacuum afford methyl 2-(isoindolin-2-ylmethyl)-4-oxo-1,4-dihydroquinazoline-5-carboxylate (**3**, 1.0 kg, 86% yield) as a white to off-white solid. To a solution of compound **3** (1 kg, 1 equiv) in ethanol (200 proof, 10 L) was added hydrazine monohydrate (2.2 L, 15 equiv). The resulting mixture was heated to 73°C and stirred at this temperature for 24 h until HPLC analysis indicated complete reaction. The mixture was cooled to room temperature and filtered. The solid was washed 3 times with ethanol (200 proof, 3 L), then dried at room tempertaure under vacuum to afford 8-(isoindolin-2-ylmethyl)-2,9-dihydro-3H-pyridazino[3,4,5-de]quinazolin-3-one (**4**, 0.kg Wt, 78% yield) as an off-white solid.

Methyl 2-(chloromethyl)-4-oxo-1,4-dihydroquinazoline-5-carboxylate (2)

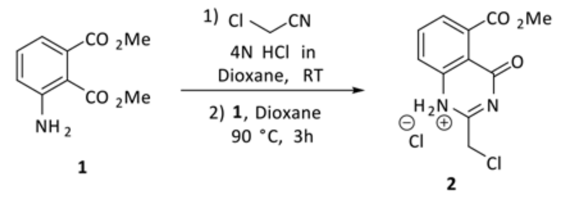

Methyl 2-(isoindolin-2-ylmethyl)-4-oxo-1,4-dihydroquinazoline-5-carboxylate (3)

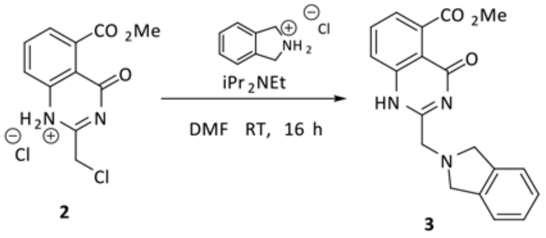

8-(isoindolin-2-ylmethyl)-2,9-dihydro-3H-pyridazino[3,4,5-de]quinazolin-3-one (4)

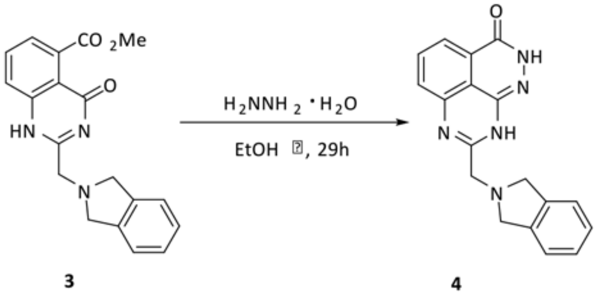

Supplementary Figure S1: Intermediates generated during chemical synthesis of E7449 (structure 4). Details on synthesis route and methodology are available in Supplementary Methods.

A

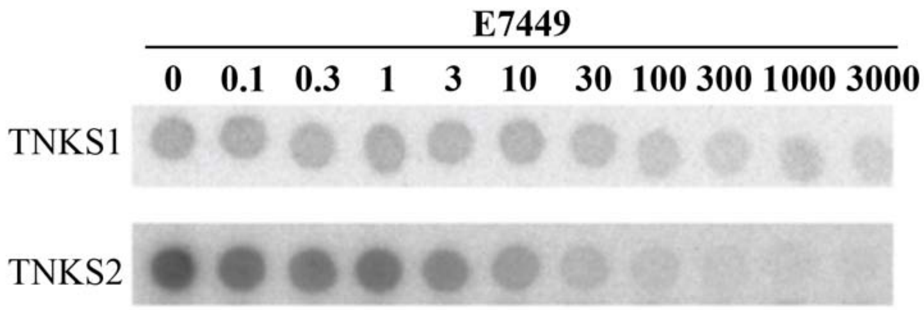

B

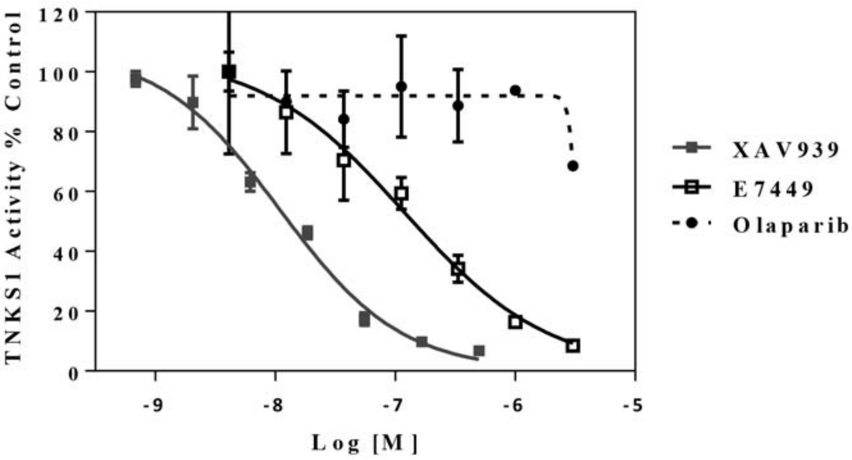

Supplementary Figure S2: A. representative image of dose-responsive E7449 inhibition of TNKS1 and 2 auto-PARylation, using ^{32}P-NAD^{+} as substrate (see materials and methods). E7449 added at 0 to 3,000 nmol/L. B. inhibition of TNKS1-mediated histone PARylation by E7449, XAV939 and olaparib. IC_{50} values were determined by non-linear regression using GraphPad Prism 5 software version 5.02.

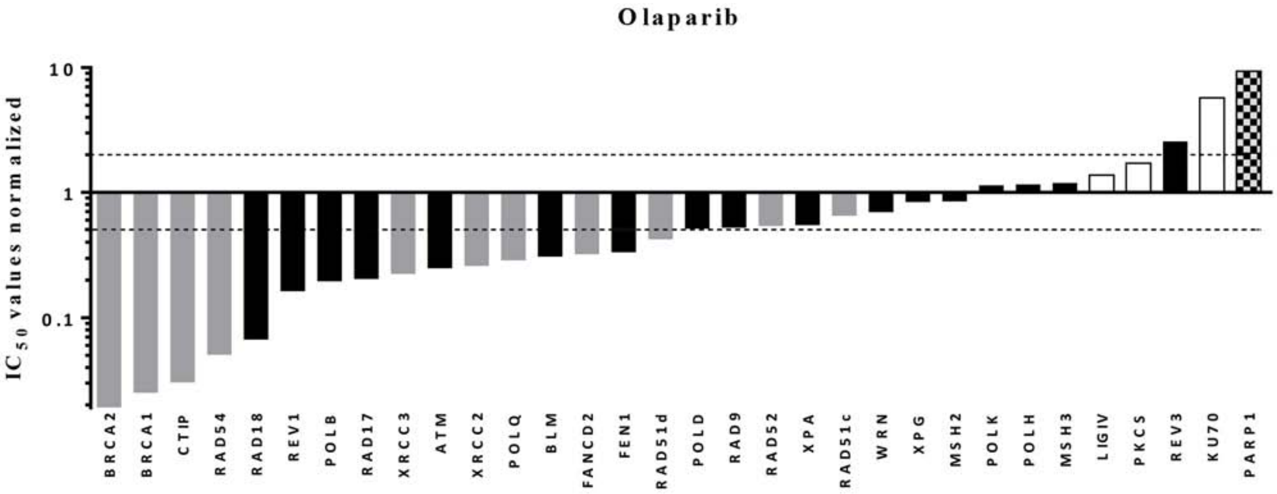

Supplementary Figure S4: Sensitivity profile of olaparib in a panel of 32 isogenic DNA repair mutant DT40 cell lines. Mean IC_{50} values from at least 3 independent assays were normalized to the IC_{50} value in wild type DT40 cells (2.1 µmol/L). Bars are shaded based on DNA repair function; checkered for PARP1, grey for HR, white for NHEJ, and black for all other DNA repair pathways. Dashed lines represent 2-fold sensitivity or resistance of cell line to olaparib versus the wild type cells.

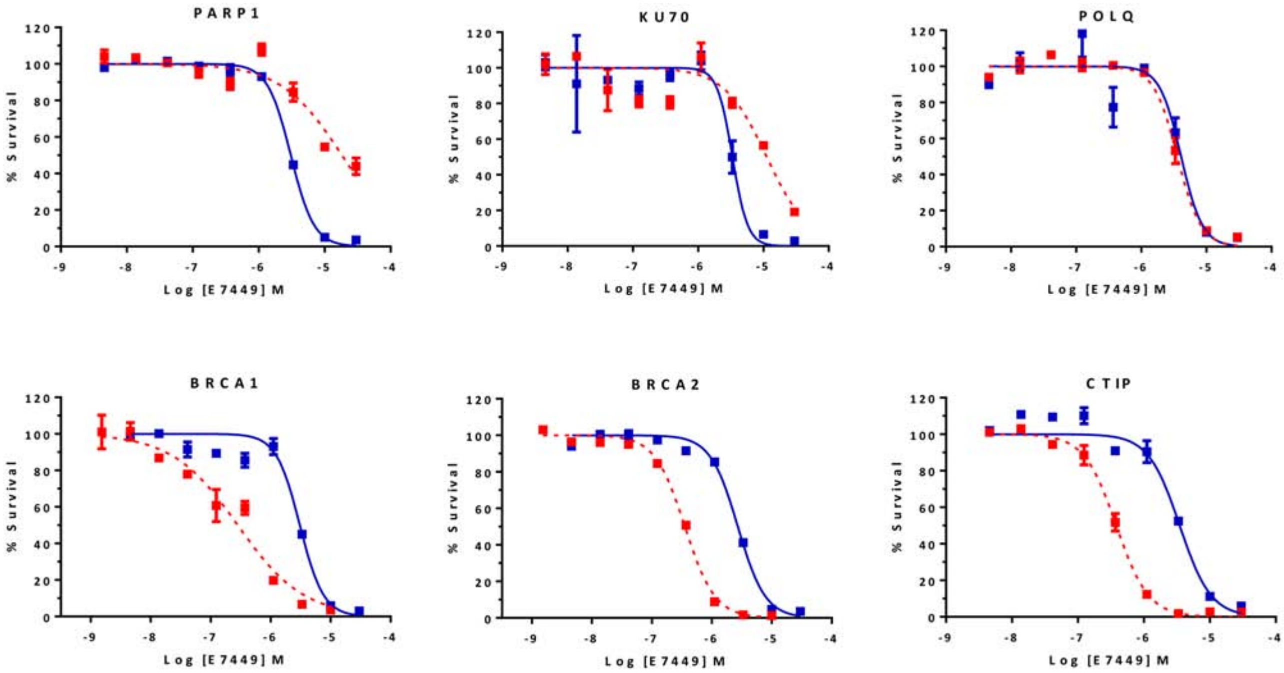

Supplementary Figure S3: Six representative graphs depicting IC_{50} curves used to generate waterfall plot in Figure 1D. E7449 was assayed in a panel of DT40 cells as outlined in the Materials and Methods. Red depicts survival of cells deficient in DNA repair proteins; PARP1, KU70, POLQ, BRCA1, BRCA2 or CTIP. Blue represents survival of wild type parent DT40 cells. IC_{50} values were calculated using the GraphPad Prism 5 software version 5.02. Loss of PARP1 or KU70 increased resistance of DT40 cells to E7449, whereas loss of BRCA1, BRCA2 or CTIP increased sensitivity to E7449, and loss of POLQ had no effect.

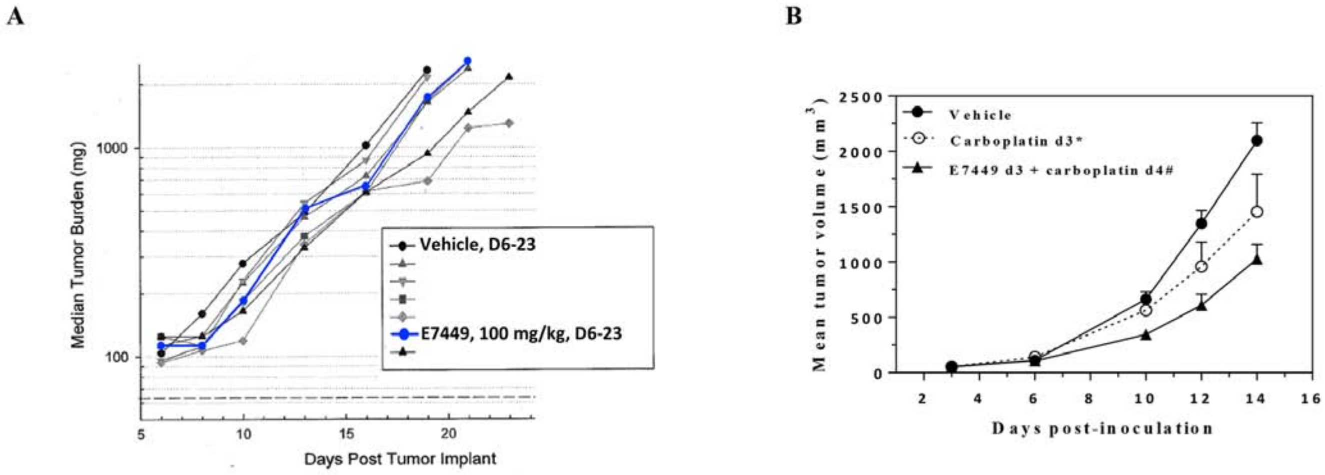

Supplementary Figure S5: Antitumor effect of E7449 or carboplatin alone or in combination in MX-1 human breast cancer xenografts. **A.** median tumor burden following treatment with E7449 at 100 mg/kg dosed once daily from day 6 to 23; E7449 as single agent was inactive. **B.** carboplatin was administered as a single i.v. dose at 60 mg/kg on day 3 as a single agent or on day 4 in combination with E7449 dosed once daily beginning on day 3, at 100 mg/kg. Data represent the mean ± SEM. Antitumor activity of carboplatin alone was modest, but statistically significant versus vehicle; *$P < 0.05$ on day 14 (student's *t*-test). Combination with E7449 enhanced the antitumor activity of carboplatin; $^{\#}P < 0.05$ versus carboplatin alone on day 14 (student's *t*-test).

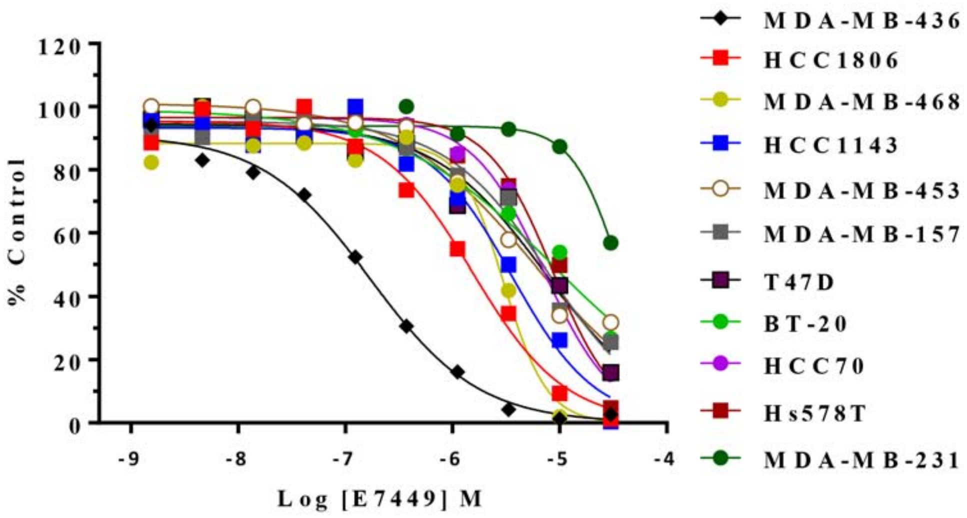

Supplementary Figure S6: Representative graph depicting IC_{50} curves used to generate waterfall plot in Figure 3A. E7449 was assayed in a panel of TNBC cells in 8 day growth inhibition assay as outlined in the Materials and Methods. IC_{50} values were calculated using GraphPad Prism 5 software version 5.02. MDA-MB-436 was the most E7449-sensitive cell line.

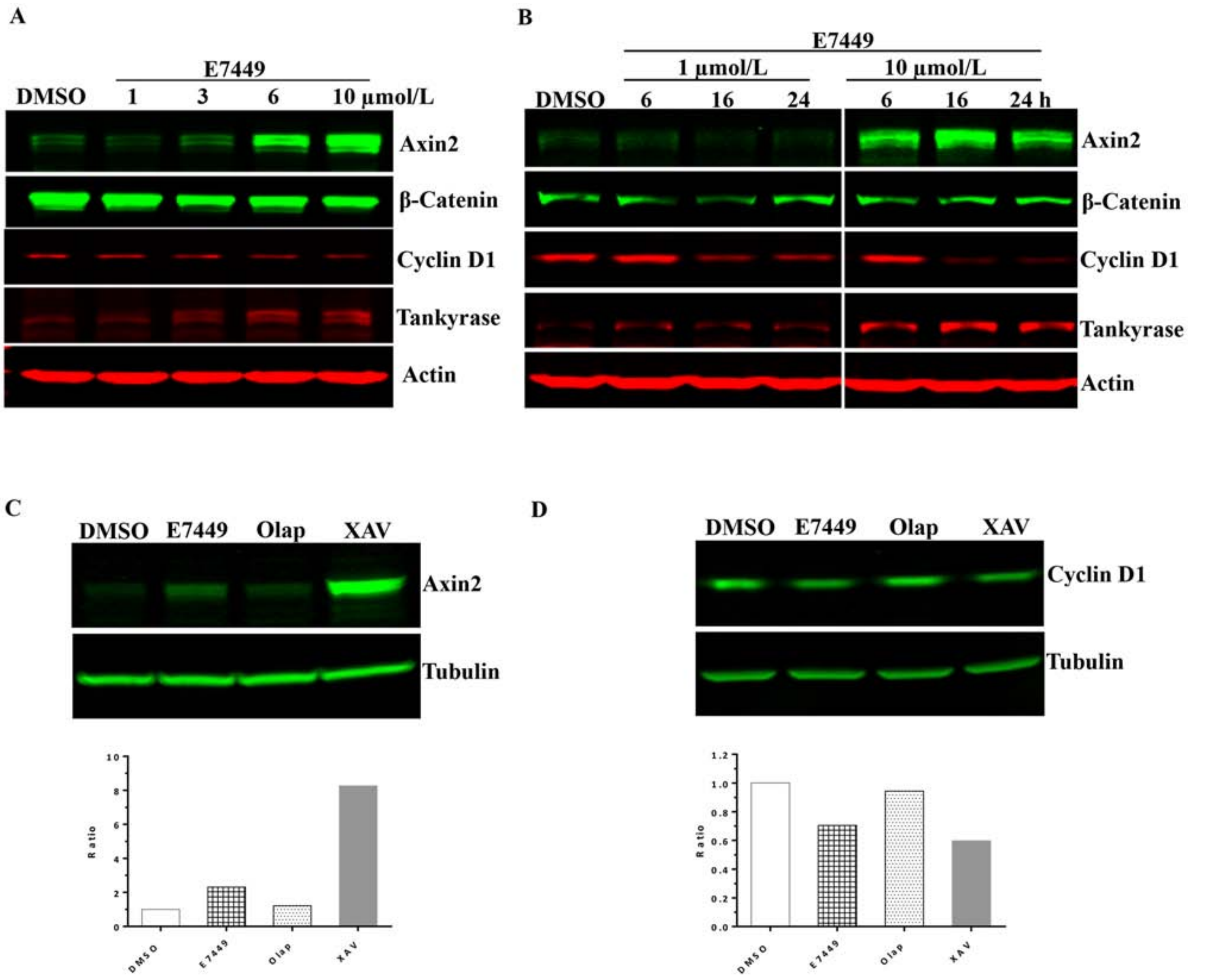

Supplementary Figure S7: E7449 inhibits Wnt signaling *in vitro*. A. dose-dependent effect of E7449 treatment on SW480 cells by western blot. Following overnight incubation of cells at indicated doses, cell lysates were probed with antibodies targeting various Wnt/β-catenin pathway proteins. **B.** time-dependent effect of E7449 treatment on SW480 cells by western blot. Cells were harvested at 6, 16 or 24 hours post-treatment and subjected to western blot analysis as above. **C.** & **D.** DLD-1 cells were treated with indicated compounds for 24 h and then subjected to western blot analysis using antibodies targeting axin2 or cyclin D1. Fluorescence intensity of bands was measured using Image Studio software on the LI-COR Odyssey imager. Ratio of analyte expression was calculated using tubulin used as a loading control.

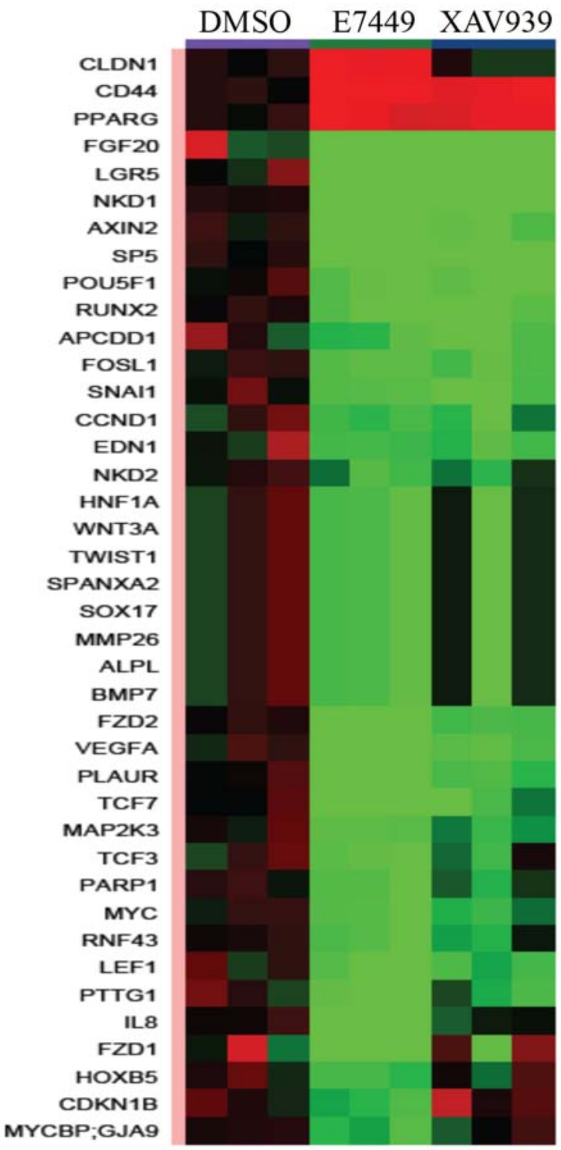

Supplementary Figure S8: Effect of E7449 treatment on expression of Wnt-related genes in DLD-1 cells. Following 72 h incubation of DLD-1 cells with E7449 or XAV939 at 30 µmol/L or DMSO, RNA was harvested and gene expression profiling performed using a custom TLDA. Expression of 40 Wnt-related genes was altered following E7449 exposure. Genes with a relative fold change of ≥ 1.5 ($P > 0.05$, student's *t*-test) versus DMSO control were subjected to hierarchical clustering (Manhattan distance) and complete linkage plotting to generate the heat map.

Supplementary Table S1:

PARP	IC_{50} nmol/L	
	Trevigen ELISA	Radioactive NAD^+
PARP1	1.0	2.0
PARP2	1.2	1.0
TNKS1	115	~50
TNKS2	ND	~50

IC_{50} values for E7449 inhibition of PARP1/2 and TNKS1/2 by various assays. Mean IC_{50} values are reported for a minimum of 3 individual assays performed in triplicate. ND = not determined.

Supplementary Table S2: Human Wnt-related genes in TLDA

Gene Symbol	Taqman Assay ID
ACTB	Hs01060665_g1
ALPL	Hs01029144_m1
APCDD1	Hs00537787_m1
ATOH1	Hs00944192_s1
AXIN2	Hs00610344_m1
B2M	Hs00984230_m1
BCL9	Hs00979216_m1
BIRC5	Hs04194392_s1
BMP7	Hs00233476_m1
BTRC	Hs00182707_m1
18S	Hs99999901_s1
CCND1	Hs00765553_m1
CD44	Hs01075861_m1
CDH1	Hs01023894_m1
CDK8	Hs00176209_m1
CDKN1B	Hs01597588_m1
CETP	Hs00163942_m1
CLDN1	Hs00221623_m1
CYR61	Hs00998500_g1
DKK1	Hs00183740_m1
EDN1	Hs00174961_m1
EFNB2	Hs00187950_m1
EGFR	Hs01076078_m1
ETS2	Hs00232009_m1
FGF18	Hs00826077_m1
FGF20	Hs00173929_m1
FGF9	Hs00181829_m1
FOSL1	Hs04187685_m1
FZD1	Hs00268943_s1
FZD2	Hs00361432_s1
FZD4	Hs00201853_m1
FZD5	Hs00258278_s1
FZD7	Hs00275833_s1
FZD9	Hs00268954_s1
GAPDH	Hs02758991_g1

(*Continued*)

Gene Symbol	Taqman Assay ID
GAST	Hs01099852_g1
GPX2	Hs01591589_m1
GREM1	Hs01879841_s1
GUSB	Hs00939627_m1
HNF1A	Hs00167041_m1
HOXB5	Hs00357820_m1
HPRT1	Hs02800695_m1
IL8	Hs00174103_m1
JAG1	Hs01070032_m1
JUN	Hs01103582_s1
KLF4	Hs00358836_m1
KLF6	Hs00810569_m1
L1CAM	Hs01109748_m1
LBH	Hs00368853_m1
LEF1	Hs01547250_m1
LGR5	Hs00173664_m1
MAP2K3	Hs00177127_m1
MAP4K1	Hs00179345_m1
MET	Hs01565584_m1
MMP2	Hs01548727_m1
MMP26	Hs00983740_m1
MMP3	Hs00968305_m1
MMP7	Hs01042796_m1
MMP9	Hs00234579_m1
MYC	Hs00153408_m1
MYCBP	Hs00429315_g1
NKD1	Hs00263894_m1
NKD2	Hs01108239_m1
NLK	Hs00212076_m1
NOS2	Hs01075529_m1
NOS3	Hs01574659_m1
NRCAM	Hs01031598_m1
PARP1	Hs00242302_m1
PAX6	Hs00240871_m1
PITX2	Hs04234069_mH
PLAUR	Hs00182181_m1

(Continued)

Gene Symbol	Taqman Assay ID
POU5F1	Hs00999632_g1
PPARD	Hs04187066_g1
PPARG	Hs01115513_m1
PTTG1	Hs00851754_u1
RNF43	Hs00993307_m1
RUNX2	Hs00231692_m1
SALL4	Hs00360675_m1
SNAI1	Hs00195591_m1
SNAI2	Hs00950344_m1
SOX17	Hs00751752_s1
SP5	Hs01370227_mH
SPANXA2	Hs03028063_gH
TCF3	Hs00413032_m1
TCF4	Hs00162613_m1
TCF7	Hs00175273_m1
TERT	Hs00972656_m1
TNKS	Hs00186671_m1
TNKS2	Hs00228829_m1
TWIST1	Hs01675818_s1
VEGFA	Hs00900055_m1
WIF1	Hs00183662_m1
WISP2	Hs00180242_m1
WISP3	Hs00365646_m1
WNT3A	Hs00263977_m1
JUP	Hs00158408_m1

Supplementary Table S3: Mouse Wnt-related genes in TLDA

Gene Symbol	Taqman Assay
Bmp7	Mm00432102_m1
Myc	Mm00487804_m1
Klf6	Mm00516184_m1
Cdh1	Mm01247357_m1
Mmp3	Mm00440295_m1
Snai2	Mm00441531_m1
Sp5	Mm00491634_m1
Mmp9	Mm00442991_m1
Map4k1	Mm01152700_m1
Tnks	Mm00558256_m1
Sall4	Mm00453037_s1
Fgf9	Mm00442795_m1
Pparg	Mm01184322_m1
Hnf1a	Mm00493434_m1
Lef1	Mm00550265_m1
Efnb2	Mm01215897_m1
Pitx2	Mm01316994_m1
Vegfa	Mm01281449_m1
Nos3	Mm00435217_m1
Mmp2	Mm00439498_m1
Car2	Mm00501576_m1
Plaur	Mm00440911_m1
Atoh1	Mm00476035_s1
Wif1	Mm00442355_m1
spike control	Ac00010014_a1
Nos2	Mm00440502_m1
Pou5f1	Mm03053917_g1
Jag1	Mm00496902_m1
Cldn1	Mm00516701_m1
Sox17	Mm00488363_m1
Ets2	Mm00468977_m1
Fzd1	Mm00445405_s1
Tcf7l2	Mm00501505_m1
Tcf7	Mm00493445_m1
Birc5	Mm00599749_m1

(*Continued*)

Gene Symbol	Taqman Assay
Krt20	Mm00508106_m1
Fzd2	Mm02524776_s1
Map2k3	Mm00435956_m1
B2m	Mm00437762_m1
Fzd9	Mm01206511_s1
Wnt3a	Mm00437337_m1
Wisp2	Mm00497471_m1
Mmp7	Mm00487724_m1
Edn1	Mm00438656_m1
Ascl2	Mm01268891_g1
Gast	Mm00439059_g1
Gapdh	Mm03302249_g1
Jun	Mm00495062_s1
Nkd2	Mm00472240_m1
18S	Hs99999901_s1
Ccnd1	Mm00432359_m1
Actb	Mm00607939_s1
Clic3	Mm00550838_m1
Tcf4	Mm00443210_m1
Grem1	Mm00488615_s1
Pax6	Mm00443081_m1
Parp1	Mm01321084_m1
Fosl1	Mm04207958_m1
Lgr5	Mm00438890_m1
Bcl9	Mm01265706_m1
Cyr61	Mm00487499_g1
Fabp2	Mm00433188_m1
Pttg1	Mm00479224_m1
Egfr	Mm00433023_m1
Fzd4	Mm00433382_m1
Tff3	Mm00495590_m1
Axin2	Mm00443610_m1
Fgf18	Mm00433286_m1
Runx2	Mm00501584_m1
Cdkn1b	Mm00438168_m1
Tnks2	Mm01196046_m1

(*Continued*)

Gene Symbol	Taqman Assay
Fgf20	Mm00748347_m1
Nrcam	Mm00663607_m1
Dkk1	Mm00438422_m1
Mycbp	Mm01192721_m1
Snai1	Mm00441533_g1
Tcf3	Mm01175588_m1
Tert	Mm00436931_m1
Fzd7	Mm00433409_s1
Ppard	Mm00803184_m1
Rnf43	Mm00552558_m1
Met	Mm01156972_m1
Fzd5	Mm00445623_s1
Tm4sf4	Mm00523755_m1
Gpx2	Mm00850074_g1
Twist1	Mm04208233_g1
Nlk	Mm00476435_m1
Vil1	Mm00494146_m1
Alpl	Mm00475834_m1
Apcdd1	Mm01257559_m1
Cd44	Mm01277163_m1
Hoxb5	Mm00657672_m1
Nkd1	Mm00471902_m1
Klf4	Mm00516104_m1
Tnfrsf19	Mm00443506_m1
L1cam	Mm00493049_m1
